# Information Sharing and Environmental Policies

**DOI:** 10.3390/ijerph7103561

**Published:** 2010-10-11

**Authors:** Fabio Antoniou, Phoebe Koundouri, Nikos Tsakiris

**Affiliations:** 1 Department of Economics, University of Cyprus, University Campus, Nicosia 1678, Cyprus; E-Mail: fabio.antoniou@ucy.ac.cy; 2 Department of Economics, University of Ioannina, University Campus, Ioannina 451 10, Greece; E-Mail: ntsak@cc.uoi.gr

**Keywords:** strategic environmental policy, pollution, emission standards, emission taxes, information sharing

## Abstract

Based on the assumption that in a standard eco-dumping model governments are uncertain about future product demand and allowing governments to obtain information from firms, we examine governments’ and firms’ incentives to share information. We show that when governments regulate polluting firms through emission standards, then governments and firms will reach an agreement concerning information sharing. The opposite holds when governments regulate pollution through emission taxes.

## 1. Introduction

Profit shifting in international firm competition is a subject that systematically attracts economists’ interest. The possibility to improve local residents’ welfare through supporting local industries *versus* foreign ones provides a channel through which superior welfare outcomes are obtained at, actually, no cost, since the policy makers do not take into account foreign residents’ welfare. In their seminal study Brander and Spencer [1] illustrated that, in the presence of international Cournot oligopolistic competition, each government faces a unilateral incentive to subsidize production of local firms and thus gain a higher market share in the common international market and thus, increase profits. This, in turn, leads to higher welfare. The disadvantage of such a rationale is that each government faces the same incentive. This means that if all policy makers subsidize the local firms then output competition is aggravated and profits fall. A prisoner’s dilemma in government competition appears.

During the last two decades World Trade Organization agreements have restricted its members from engaging in such a behavior, but the unilateral incentive to increase the market share of exporting firms remains in place. A voluminous literature referred to as “strategic environmental policy literature” examines how environmental policy instruments can be used, in the presence of environmental externalities, as second best instruments for international trade purposes when traditional trade taxes, subsidies and quotas are prohibited or restricted. Specifically, in the context of international oligopolistic competition and under complete information, among others, [2–7] conclude that when firms compete in outputs, the governments, in their effort to enhance the international competitiveness of local exporting firms, have a unilateral incentive to pursue laxer environmental policies, *i.e.*, use of lax emission standards or emission taxes (empirical support concerning ecological dumping can be found in [8–11]). In general, there are two ways to regulate industrial pollution: (a) through the use of quantity constraints, which translate into several forms of maximum emission standards or pollution permits; and (b) through emission taxes.

A common assumption of these studies is that governments and firms act in a complete information environment. This means that governments might perfectly foresee the future market conditions or the costs of the firms. Nonetheless, this assumption is not innocuous. As clearly indicated in the seminal study of Weitzman [12], when a regulator is uncertain about marginal abatement cost and damage functions there is always a loss in terms of welfare as the *ex ante* optimal regulation is different from the *ex post* one. Hence, in order to select the optimal policy instrument the welfare losses must be compared. Nannerup [13] claims that in a strategic environmental policy setting the presence of incomplete information might reduce the prisoner’s dilemma. In other words, when the governments are uncertain, but at the same time they can set a screening mechanism, then environmental regulation is closer to the Pigouvian level compared to the complete information scenario.

It is clear from these studies that information plays a key role. Creane and Miyagiwa (CM) using a strategic trade model under incomplete information recognized the possibility that governments and firms might share information as this is mutually beneficial [14]. The authors argue that this is the case when the firms compete a là Cournot. Indeed the U.S. Export-Import Bank requires detailed information both for the demand and the cost of the industry. A specific example is the subsidization of the aircraft industry which only occurs when the industry provides the government with the operating statistics for at least the past three years of operation. Contrary to that, when the firms compete a là Bertrand, then agreement between firms and governments is no longer viable as the firms prefer to keep their private information. These results hold regardless of the mode of uncertainty, *i.e.*, demand or cost. At the same time the authors recognize that under demand uncertainty and quantity competition it appears an informational prisoner’s dilemma, where the governments and the firms share information despite the fact that they would be better off if they would not. Hence, they identify another channel through which welfare of the exporting countries might be harmed when the governments cannot achieve a cooperative solution. The aim of the current study is to examine whether governments and firms have the incentive to share information about demand when this is private to the firms, in a strategic environmental policy setting. Instead of examining the two polar cases regarding the mode of competition we study two alternative scenarios regarding the mode of regulation, while keeping fixed the assumption of Cournot competition (in ecological dumping literature regulation is set below the Pigouvian level only in the case where firms compete in quantities). Assuming, initially, that the governments select emission standards to control pollution we show that the governments and the firms, similarly to CM, agree to share information. Contrary to CM, however, now, the informational prisoner’s dilemma disappears. Putting it differently, both governments and firms are better off when they do agree to share information. The main value added of this study, however, is obtained for the case where governments select emission taxes to deal with pollution. Then, we illustrate that the participants will not share information as the firms are unwilling to do so. Hence, the mode of the policy instrument chosen might affect the economy’s informational structure. In terms of real world relevance, the EU has modernized European chemicals legislation and established REACH, which is an integrated system for the registration, evaluation, authorization and restrictions of chemicals. In order to sustain this program the EU has set up the European Chemicals Agency. Hence, given the selected information from various industries the regulator determines the restrictions applied to the industry (see Regulation (EC) No 1907/2006 of the European Parliament).

The structure of the paper is as follows. In Section 2 the model is introduced. Then, in Sections 3 and 4 the cases of emission standards and taxes are presented and solved respectively. Finally, Section 5 provides some concluding remarks. All proofs are relegated to an Appendix.

## 2. The Model

We consider a symmetric two country (home and foreign) international duopoly model, where each firm belongs to a different country and produces a homogenous good whose consumers reside in a third country. Consumers preferences can be mapped into a quasi-linear utility function which implies a linear inverse demand of the form *p* = *B* − *x* − *X* + *θ*, where B is the demand intercept, x, X are the output levels for the domestic and the foreign firm respectively and θ is known by the firms and not the governments. Following the relevant literature [14,15] θ can be faced as a random variable reflecting any possible positive and negative additive shocks in demand and is assumed to follow a distribution with mean zero (throughout the paper the foreign country’s variables and functions are indicated with upper case letters. Due to assumed symmetry, the comparative statics analysis is carried out primarily in terms of home country variables. Furthermore, uncertainty is introduced in a way that the results obtained are comparable to the ones in the relevant literature. We assume that when θ takes negative values, interior solutions for our variables are still obtained).

Both firms face the same technology which implies that a unit of production generates a unit of pollution (z). However, an exogenous abatement technology is assumed to exist and thus net pollution equals production minus abatement carried out by the firm:

(1) z=x−a

The abatement cost function is assumed to be convex of the form:

(2) $$c_{a}=\frac{1}{2}{\text{ga}}^{2}$$

where g is a positive scalar which determines the cost of pollution control. The profit function of the domestic firm depends on the policy instrument chosen by the government in order to regulate pollution and is given by the following expression:

(3) $$\pi =(B-x-X+\theta )x-\text{cx}-c_{a}-\text{tz}$$

where c is marginal cost of production (common for both firms and implies constant returns of scale) and tz are the tax payments due to pollution when a tax is the policy instrument chosen. The choice variables of the firms are output and abatement level.

Regulation of pollution by the governments takes place prior to production decisions. We examine two different ways to regulate pollution. First, we assume that governments can use an emissions standard, *i.e.*, a maximum allowed level of pollution by the firms. Additional emissions must be abated by the firm. Hence, emissions generated by the firm, z, must coincide with the standard set by the government which results as a quantity constraint (note that standards and pollution permits are equivalent policy instruments only in the case where the latter are non-tradable. If the opposite holds, then the equivalence breaks down. Here we allow for standards or non-tradable permits since the existence of tradable permits would demand a strategic analysis among the firms in the permits market which is out of the scope of this paper). The alternative policy instrument available to the governments is a tax for each unit of emissions, t, which is considered as a price constraint. Governments in both regimes choose the optimal level of regulation by maximizing welfare which is given by:

(4) w=π+tz−d

where tz are the revenues from the pollution tax when this is implemented and d stands for the damage caused from pollution and has the following form:

(5) $$d=\frac{1}{2}{\text{kz}}^{2}$$

where z is domestic pollution. The coefficient k is positive and determines the injuriousness of the pollutant (if we allow pollution to be trans-boundary, *i.e.*, *d* = ½*k*(*z* + *γZ*)^2^, where 0 < *γ* ≤ 1, the results do not change qualitatively).

**Figure 1.**
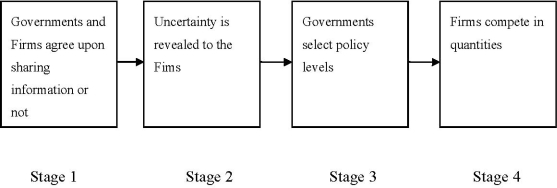
Time Structure of the Game.

Before any decision takes place we assume that the government and the firm in each country may agree to share information (the governments are unable to obtain information through an alternative channel, e.g., through a study, as the demand function is determined in a third country). This is the case if and only if both participants agree. If the government or the firm is harmed by such an agreement they refuse participation. Following the assumptions above we summarize the time structure of the game in Figure 1.

Initially, in Stage 1, the firms decide whether they are willing to disclose information or not and at the same time the governments decide whether they will accept it or not. If they both agree, then they create an institutional structure such that information disclosure is verifiable through setting a prohibitive penalty cost for those who do not comply. We assume that the set up cost of an agreement is negligible. Then, in Stage 2 uncertainty is revealed to the firms. Given that, in Stage 3, the governments select the level of regulation (taxes or standards) in order to regulate pollution. Finally, in Stage 4, the firms choose quantities so as to maximize their profits.

## 3. Emission Standards

In order to determine whether a government will agree with the corresponding firm to share information or not, we derive the Nash equilibria of the game for all the possible scenarios. In other words, we complete the full payoff matrices of expected welfare levels and profits for the domestic government and firm respectively, for every possible contingency, given that the rival partners share information or not.

### 3.1. Information Sharing

Initially, we assume that the governments and the firms in the two countries agree to share information. Hence, the problem reduces to a simple complete information game. To derive the Subgame Perfect Nash equilibrium we solve the problem via backwards induction. When standards are used as an instrument, firms have a unique control variable (production), since abatement must be chosen such that equation (1) is satisfied. Bearing this in mind, we maximize domestic profits with respect to output and obtain the reaction function of output (*x^R^*):

(6) $$\begin{array}{l} \;\;\;\frac{d\pi }{dx}=0\\ s.t.a=x-z\\ \;\;\;\;\;\Leftrightarrow x^{R}=\frac{B-c+\theta -X^{R}+\text{gz}}{2+g} \end{array}$$

where $\frac{\partial x^{R}}{\partial X^{R}}=-\frac{1}{2+g}<0$ is the slope of the domestic firm’s reaction function. Solving simultaneously the domestic and the analogue foreign firms’ reaction functions we obtain equilibrium outputs as a function, among other things, of standards:

(7) $$x=\frac{(B-c+\theta )(1+g)+g(2+g)z-\text{gZ}}{\left(1+g)(3+g\right)}$$

From equation (7) and the respective foreign equilibrium output we obtain that $\frac{dx}{dz}>0$, $\frac{dX}{dZ}>0$, $\frac{dx}{dZ}<0$ and $\frac{dX}{dz}<0$. The last two derivatives imply that when regulation abroad is relaxed, local output falls due to the negative slope of the reaction function (6). This derivative is the core of the so called “strategic environmental policy” literature (see [2–7]), since it creates incentives for the governments to relax regulation in order to favor, *i.e.*, shift profits to their own exporting firms.

Given equilibrium outputs, governments select the optimal level of emission standards by maximizing welfare:

(8) $$\begin{array}{l} \frac{dw}{dz}=\underset{\begin{array}{l} \text{zero}\;\text{due}\;\text{to}\\ \;\;\;\;\;\text{F}\text{.O}\text{.C} \end{array}}{\underset{︸}{\frac{\partial \pi }{\partial x}\frac{\partial x}{\partial z}}}+\underset{\begin{array}{l} \text{direct}\\ \text{effect}\\ \;(+) \end{array}}{\underset{︸}{\frac{\partial \pi }{\partial z}}}+\underset{\begin{array}{l} \text{strategic}\\ \;\;\;\text{effect}\\ \;\;\;\;(+) \end{array}}{\underset{︸}{\frac{\partial \pi }{\partial X}\frac{\partial X}{\partial z}}}-\underset{\begin{array}{l} \text{regulation}\\ \;\;\text{benefit}\\ \;\;\;\;\;(+) \end{array}}{\underset{︸}{\frac{\partial d}{\partial z}}}=0\\ \;\;\;\;\;\Leftrightarrow z=\frac{g{\left(2+g\right)}^{2}[(B-c+\theta )(1+g)-gZ]}{\zeta_{1}} \end{array}$$

where *ζ*_1_ = *g* {9 + 2*g* [8 + *g*(5 + *g*)]} + (1 + *g*)^2^ (3 + *g*)^2^ *k* (since the problem is concave we neglect the second order conditions). Equation (8) gives the reaction function of the domestic regulator. That is, *δz/δZ* < 0 implies that domestic and foreign emission standards are strategic substitutes [16]. If the foreign regulator tightens its standard then the domestic one relaxes its own and *vice-versa*. Strategic substitutability of standards follows when, for example, the standard is tighter in a foreign location, thus, production in that country falls, which in turn increase the production of the home firm through its output reaction function in (6). As a result, the home’s firm marginal cost of abatement (direct effect) and the regulator strategic incentive (strategic effect) increase, which in turn force the regulator in home to relax further the standard.

Solving simultaneously Stage 4 equilibrium output (7), the domestic government’s reaction function (8) and the corresponding equations for the foreign firm and government we obtain the Subgame Perfect Nash equilibrium:

(9) $$\left\{\begin{array}{l} x^{cc}=X^{cc}=\frac{\left(B-c+\theta )(1+g)(3+g)(g+k\right)}{\zeta_{2}}\\ \;\;\;\;z^{cc}=Z^{cc}=\frac{(B-c+\theta )g{\left(2+g\right)}^{2}}{\zeta_{2}} \end{array}\right\}$$

where *ζ*_2_ = *g*[9 + *g*(11 + 3*g*)] + (1 + *g*)(3 + *g*)^2^ *k* and the superscript (cc) indicates that governments and firms in both countries share information. These are the equilibrium levels of outputs and standards. In case the demand is high (θ is high) then both outputs and standards are high and vice-versa. To determine profits and welfare we substitute equilibrium values given in (9) in (3) and (4) respectively (all the calculations in the paper were done using Mathematica 6):

$$\begin{array}{c} \pi^{cc}=\frac{{\left(B-c+\theta \right)}^{2}[(2+g)(g+k)\zeta_{1}+g^{2}{\left(2+g\right)}^{4}k]}{2\zeta_{2}^{2}}\\ \text{and}\;w^{cc}=\frac{{\left(B-c+\theta \right)}^{2}(2+g)(g+k)\zeta_{1}}{2\zeta_{2}^{2}} \end{array}$$

In Stage 1 of the game from the governments perspective θ is unknown and thus the expected profits and welfare are:

(10) $$E[\pi^{cc}]=\frac{\left[{\left(B-c\right)}^{2}+\text{var}(\theta )][(2+g)(g+k)\zeta_{1}+g^{2}{\left(2+g\right)}^{4}k\right]}{2\zeta_{2}^{2}}$$

(11) $$\text{and}\;E[w^{cc}]=\frac{[{\left(B-c\right)}^{2}+\text{var}(\theta )](2+g)(g+k)\zeta_{1}}{2\zeta_{2}^{2}}$$

where var(θ) is the mean-preserving spread distribution (variance) of the demand intercept. We observe that *ex ante* profits (10) and *ex ante* welfare (11) depend positively on var(θ). This is due to the convexity of the profit function with respect to the demand intercept. Hence, as the variability of θ increases the expected values of profits and welfare also increase.

### 3.2. No Information Sharing

Now, we examine the scenario where the firms and the governments do not share information. If this is the case then the governments act under incomplete information as θ is unobservable for them. Thus, the equilibrium notion that we use is Bayes Nash equilibrium. Firms’ maximizing problem follows in the lines of the previous analysis, while welfare maximization follows a slightly moderated one. Since θ is unobservable to the governments, yet they know the distribution that it follows, they maximize their expected welfare with respect to the emission standard. This results to an equivalent reaction function given in (8) after setting θ = 0.

Solving simultaneously (7) and (8) after setting θ = 0 as well as the corresponding equations for the foreign firm and government we obtain the Bayes Nash equilibrium:

(12) $$\left\{\begin{array}{c} x^{nn}=X^{nn}=\frac{\left(B-c)(1+g)(3+g)(g+k\right)}{\zeta_{2}}+\frac{\theta }{3+g}\\ z^{nn}=Z^{nn}=\frac{(B-c)g{\left(2+g\right)}^{2}}{\zeta_{2}} \end{array}\right\}$$

where the superscript (nn) represents the fact that the governments and the firms do not agree to share information. Abatement can be calculated through equation (1). As θ is unobservable by the governments, it follows that in equilibrium, contrary to output and abatement, emission standards do not depend on θ. Moreover, the strategic effect is positive and creates an incentive to relax own regulation, *i.e.*, increase z, compared to the first best case where regulation is set such that the marginal cost of abatement and the marginal damage from pollution are equated, *i.e.*, *δπ/δz* = *δd/δz*, thus the externality is fully internalized and then, the strategic effect is zero. In order to determine the level of expected profits and welfare in the case of standards we substitute the equilibrium values given in (12) and the implied abatement level by (1), into (3) and (4) respectively. Taking expectations and after some algebraic manipulation we get:

(13) $$E[\pi^{nn}]=\frac{{\left(B-c\right)}^{2}(2+g)[g\zeta_{1}+{\left(1+g\right)}^{2}{\left(3+g\right)}^{2}k(g+k)]}{2\zeta_{2}^{2}}+\frac{\left(2+g\right)}{2{\left(3+g\right)}^{2}}\text{var}(\theta )$$

(14) $$\text{and}\;E[w^{nn}]=\frac{{\left(B-c\right)}^{2}(g+k)(2+g)\zeta_{1}}{2\zeta_{2}^{2}}+\frac{\left(2+g\right)}{2{\left(3+g\right)}^{2}}\text{var}(\theta )$$

The second right hand side terms of (13) and (14) indicate that expected profits and welfare depends positively on var(θ), *i.e.*, *ex ante* profits and welfare increase with uncertainty. This is true because the firms select outputs after θ is revealed. From a technical aspect two opposing effects determine this outcome. A positive effect is due to the convexity of the profit function in terms of the demand intercept and it is similar to the ones introduced by Cooper and Riezman [15] and CM in the context of strategic trade models. Since pollution is fixed at the selected level, the damage from pollution is not affected by the demand variability. A negative effect, absent from the strategic trade models, is attributed to the convexity of the abatement cost function, which implies that high var(θ) entails a negative impact on expected profits and thus welfare. Nonetheless, the positive effect is stronger than the negative one and thus, the overall effect remains positive.

### 3.3. Information Game

In order to move in Stage 1 of the game and examine whether the firms and the governments will share information or not, we need to solve for the asymmetric cases as well, where the partners in one country agree to share information, while the rival pair does not and *vice-versa*. Due to similarity with the analysis thus far we relegate the solutions of the asymmetric case in the Appendix. Now, having derived the expected profits and welfare levels for every possible contingency, we are ready to determine the Nash equilibrium of the game.

Before doing so we provide the optimal strategy of the domestic regulator and the firm for each possible combination of information sharing chosen by the rival pair. Lemma 1 summarizes the optimal strategy for the domestic pair (the optimal strategies for the foreign firm and government are directly implied by the ones of their correspondents in the home country).

**Lemma 1** *When emission standards are the policy instrument in use, then with unknown common demand:*

*It is a dominant strategy that the firm and the government share information regardless of what the rival pair does, i.e.*, {*E*[*w^cc^*] > *E*[*w^nc^*], *E*[*π^cc^*] > *E*[*π^nc^*]}*and* {*E*[*w^cn^*] > *E*[*w^nn^*], *E*[*π^cn^*] > *E*[*π^nn^*]}.

Proof in Appendix

Using Lemma 1, we define the Nash equilibrium of the information sharing game in the following proposition:

**Proposition 1** *When emission standards are the policy instrument in use, then with unknown common demand:*

#### In the Nash equilibrium each pair agrees to share information

Proposition 1 states that as it is a dominant strategy for the governments and the firms to share information it is also a Nash equilibrium of the game. The benefits from sharing information are greater than the losses. In particular, the benefits for the firms and the governments from sharing information arise from the convexity of the profit functions with respect to the demand intercept. The losses are attributed to the convexity of the damage function of pollution with respect also to the demand intercept. When the firms decide to share their information with the governments then in exchange they get laxer regulation (higher standards) as standards depend positively on θ [see equation (9)] in good times, which in terms of our modeling implies times of high demand, while when demand is low then regulation is tighter. Thus, when demand is high, the firms face even lower abatement costs because the binding level of emissions is relaxed and *vice-versa*. At the same time when regulation is laxer the firm is more aggressive in international competition gaining a larger market share and higher rent shifting from the rival firm. Hence, the mechanism which supports laxer regulation as a commitment device over the rival firm is re-enforced benefiting both the government and the firm. Although at times of lower demand the opposite holds, the losses suffered in this case are lower than the gains at times of higher demand. Therefore, adjusting standards to demand makes the welfare and the profit functions more convex and in turn increase their levels. It is also important to note that the validity of this argument does not depend on whether the rival pair shares information or not. The expected levels of profits and welfare always depend positively on the variance of the demand intercept.

Given this result which so far parallels the one of CM, although in a different context, it is interesting to check if the result of sharing information is socially desirable. This is true when the expected welfare level in the sharing information case is higher compared to the case where none of the two pairs share information. Proposition 2 summarizes this comparison:

**Proposition 2** *When emission standards are the policy instrument in use, then with unknown common demand:*

*Expected welfare under information sharing is higher compared to the corresponding one where the governments do not receive information, i.e*., *E*[*w^cc^*] > *E*[*w^nn^*].

Proof in Appendix

This result is of major importance since it states that when emission standards are the unique policy instrument in use, information sharing occurs and this is superior in terms of expected welfare compared to the case where the two pairs do not reach an agreement. Put it differently, from the social perspective the Nash equilibrium is socially optimal. At the same time it can be shown that each firm and government prefer that the rival pair do not share information regardless of their agreement (the proof of this claim is neglected for brevity and it can be provided upon request by the authors). If the domestic players share information, then the domestic firm and government are better off if the rival pair do not reach an agreement. In this scenario, the domestic firm faces more flexible standards which in turn, when demand is high, allow the domestic firm to obtain an even larger market share, while in the opposite case the market share shrinks. The benefits attributed to the convexity of the profit function with respect to the demand intercept are now higher. If the domestic pair do not share information they prefer that the rival pair does the same. If not, then at times of high demand the rival government indirectly subsidizes the corresponding firm through laxer regulation shrinking the market share of the domestic firm and reducing so its expected profits. Contrary to that, when demand is lower then regulation is stricter benefiting the domestic pair who decided to not share information. However, the first outcome prevails to the second one. The fact that each pair prefers that the rival pair is not informed does not lead to an “informational prisoner’s dilemma” as CM claim in their model. Put it differently, when emission standards are used to subsidize exports instead of subsidies, information sharing leads to a superior outcome in terms of expected welfare. It is interesting that even if both pairs prefer that the rival one does not, indeed they do share information and this is mutually beneficial compared to the case where they do not. The benefits arising from the convexity of the profit function with respect to the demand intercept when the two pairs reach an agreement outweigh the expected welfare losses attributed to the variability of standards and, thus, the variability in the damage from pollution.

## 4. Emission Taxes

### 4.1. Information Sharing

In contrast to the previous case we now assume that both governments use taxes to control pollution. Now firms have two control variables available, output and the abatement level. Solving backwards we derive the first order conditions for the domestic firm:

(15) $$\frac{d\pi }{dx}=0\Leftrightarrow x=\frac{B-c-t+\theta -X}{2}$$

(16) $$\frac{d\pi }{da}=0\Leftrightarrow a=\frac{t}{g}$$

The output reaction function of the domestic firm is implied by equation (15). We observe that when taxes are used the output reaction function is steeper than the corresponding one in the case of standards. That is because the use of a standard implies a positively sloped total marginal cost of production (*i.e.*, marginal cost plus marginal cost of abatement) due to the existence of the maximum standard constraint, while implementation of a tax implies horizontal total marginal cost of production (*i.e.*, marginal cost of production plus the tax) as it increases proportionally to the tax. It follows that given a change in the output decisions of the rival firm, the responsiveness of the home firm’s output is greater when a tax rather than a standard is implemented. The profit maximizing condition with respect to abatement is given by equation (16) and states that the marginal cost of abatement equals the pollution tax. Equation (16) is used such that *a* does not appear into the profit function. The equilibrium values of outputs as a function of taxes are obtained by solving the domestic and foreign firms’ reaction functions simultaneously:

(17) $$x=\frac{B-c+\theta -2t+T}{3}$$

Examining the domestic government’s decision about the optimal tax we maximize welfare with respect to the emissions tax. Thus, for home we have:

(18) $$\begin{array}{l} \frac{dw}{dt}=\frac{\partial \pi }{\partial x}\frac{\partial x}{\partial t}+\frac{\partial \pi }{\partial t}+\frac{\partial \pi }{\partial z}\frac{\partial z}{\partial t}+\frac{\partial \pi }{\partial X}\frac{\partial X}{\partial t}+\frac{\partial tz}{\partial t}-\frac{\partial d}{\partial z}\frac{\partial z}{\partial t}=0\\ \;\;\;\;\Leftrightarrow t=\frac{g[3k+g(-1+2k)](B-c+T+\theta )}{\tau_{1}} \end{array}$$

where *τ*_1_ = *g*(9 + 4*g*) + (3 + 2*g*)^2^ *k*. If 3*k* + *g*(−1 + 2*k*) > 0 then the reaction function of the domestic regulator implies that taxes are strategic complements, which as we will see later is a sufficient condition for the existence of an interior solution in equilibrium, otherwise we obtain a negative pollution tax.

In order to obtain the equilibrium levels of outputs, taxes and pollution in the two countries we solve simultaneously equations (1), (16), (17), (18) and their analogues for the foreign firm and government to obtain:

(19) $$\left\{\begin{array}{c} x_{t}^{cc}=X_{T}^{cc}=\frac{\left(B-c+\theta )(3+2g)(g+k\right)}{\tau_{2}}\\ z_{t}^{cc}=Z_{T}^{cc}=\frac{\left(B-c+\theta )2g(2+g\right)}{\tau_{2}}\\ t^{cc}=T^{cc}=\frac{\left(B-c+\theta )g[3k+g(-1+2k)\right]}{\tau_{2}} \end{array}\right\}$$

where *τ*_2_ = *g*(9 + 5*g*) + (3 + *g*)(3 + 2*g*)*k* and the subscripts (t) and (T) denote that taxes are implemented as a policy instrument. As already mentioned, $k>\frac{g}{3+2g}$ is a sufficient condition for the existence of an interior solution. We observe that output is more sensitive to demand variability when taxes are implemented instead of standards. This is due to the fact that total marginal cost of output is steeper when a standard is used in comparison to the case of a tax where marginal cost is flat. Hence, firms are more flexible in the case of taxes. The important feature that arises from the implementation of taxes is that, now, in times of high demand the tax rises which implies a tighter environmental policy and vice-versa. Contrary to the case of standards, now the government does not indirectly subsidize the firm for sharing information. If the firm shares its private information about the demand, it will be taxed further if demand is higher than expected or it will face a tax cut in case the demand lies below the expected level. This result is crucial and drives the results of the paper.

Substituting the new equilibrium levels in equations (3) and (4) we obtain profits and welfare levels for each country:

$$\begin{array}{c} \pi_{t}^{cc}=\frac{{\left(B-c+\theta \right)}^{2}(2+g)[g^{2}(9+8g)+6g(3+2g)k+{\left(3+2g\right)}^{2}k^{2}]}{2\tau_{2}^{2}}\\ \text{and}\;w_{t}^{cc}=\frac{{\left(B-c+\theta \right)}^{2}(2+g)(g+k)\tau_{1}}{2\tau_{2}^{2}} \end{array}$$

As in the case of standards, we the ex ante values for profits and welfare as follows:

(20) $$E[\pi_{t}^{cc}]=\frac{\left[{\left(B-c\right)}^{2}+\text{var}(\theta )](2+g)[g^{2}(9+8g)+6g(3+2g)k+{\left(3+2g\right)}^{2}k^{2}\right]}{2\tau_{2}^{2}}$$

(21) $$\text{and}\;E[w_{t}^{cc}]=\frac{[{\left(B-c\right)}^{2}+\text{var}(\theta )](2+g)(g+k)\tau_{1}}{2\tau_{2}^{2}}$$

From equations (20) and (21) we observe that the expected values of profits and welfare depend positively on var(θ). The explanation for this outcome lies between the lines of the corresponding case of standards. The profit function is a convex function with respect to the demand intercept yielding a risk lover behavior by the firms. Despite the fact that the tax act as an automatic stabilizer any shock in the demand still affects the output.

### 4.2. No Information Sharing

In case that the governments and the firms do not reach an agreement the governments act under incomplete information. Firms’ maximizing problem remains the same as in the complete information case, while welfare maximization is moderated. Now, the two governments maximize their expected welfare with respect to the emission standard. Welfare maximization by the domestic government yields a reaction function given in (18) after setting θ = 0.

Solving simultaneously (1), (16), (17) and (18) after setting θ = 0 as well as the corresponding equations for the foreign firm and government we obtain the Bayes Nash equilibrium:

(22) $$\left\{\begin{array}{c} x_{t}^{nn}=X_{T}^{nn}=\frac{\left(B-c)(3+2g)(g+k\right)}{\tau_{2}}+\frac{\theta }{3}\\ z_{t}^{nn}=Z_{T}^{nn}=\frac{\left(B-c)2g(2+g\right)}{\tau_{2}}+\frac{\theta }{3}\\ t^{nn}=T^{nn}=\frac{\left(B-c)g[3k+g(-1+2k)\right]}{\tau_{2}} \end{array}\right\}$$

Comparing the solutions given in (22) and (19), several inferences can be drawn that play a significant role in determining the expected national welfare levels. When governments are not informed, the level of emission taxes in each country is determined at a specific level and it is not affected by θ. Contrary to the situation where the governments and the firms share information the governments do not adjust their policy to θ and, thus, when θ is positive the firm may adjust its output without being penalized by the government. This, together with the fact that now abatement does not depend on θ, creates a clear disincentive to the firms to reveal their private information.

However, this is not true for the governments. If we compare the level of pollution in equilibrium in the two polar cases we obtain that pollution is higher in the incomplete information case when θ is positive and lower if θ has the opposite sign (the difference of the two is given by: $z_{t}^{nn}-z^{nn}=\frac{(3+g)[3k+g(-1+2k)]\theta }{\tau_{2}}$. It is apparent that the sign of the difference depends on the sign of θ). This means that the variability of pollution is higher in the incomplete information case. This is expected to harm the governments in terms of expected welfare as pollution enters in the damage function which in turn affects welfare negatively. Therefore, when a government decides to obtain information from the firm for the current status of the demand needs to weight the two opposing effects. On the one hand the positive effect is sourcing from the lower variability in pollution and, on the other hand, the negative effect reflecting the lower expected profits. Which of the two prevails is not clear and needs to be examined in detail.

To determine the level of expected profits and welfare for this scenario we substitute the equilibrium values given in (22) and the implied abatement level in (16), into (3) and (4) respectively. Taking expectations and after some algebraic manipulation we get:

(23) $$E[\pi_{t}^{nn}]=\frac{\left[{\left(B-c\right)}^{2}](2+g)[g^{2}(9+8g)+6g(3+2g)k+{\left(3+2g\right)}^{2}k^{2}\right]}{2\tau_{2}^{2}}+\frac{1}{9}\text{var}(\theta )$$

(24) $$\text{and}\;E[w_{t}^{nn}]=\frac{{\left(B-c\right)}^{2}(2+g)(g+k)\tau_{1}}{2\tau_{2}^{2}}-\frac{\left(k-2\right)}{18}\text{var}(\theta )$$

It is clear from (23) that expected profits depend positively on var(θ). From equation (24), however, it is not clear if expected welfare depends positively or negatively on var(θ). If k < 2, expected welfare depends positively on the variance of the demand intercept. In this case the damage caused from pollution is not severe enough. The extra variability of pollution is not sufficient to reverse the positive sign of var(θ). Apparently, the opposite holds when k > 2.

### 4.3. Information Game

To complete the full payoff matrix, the asymmetric cases and the expected values of profits and welfare must be calculated (see Appendix). Given these, we provide the optimal response of the domestic regulator and the firm for each possible combination of information sharing chosen by the rival pair. Lemma 2 summarizes these results:

**Lemma 2** *When emission taxes are the policy instrument in use, then with unknown common demand:*

*It is a dominant strategy for the government to accept information and for the firm to not share information regardless of what the rival pair does, i.e.,* { $E[w_{t}^{cc}]>E[w_{t}^{nc}]$, $E[\pi_{t}^{cc}]<E[\pi_{t}^{nc}]$} *and* { $E[w_{t}^{cn}]>E[w_{t}^{nn}]$, $E[\pi_{t}^{cn}]<E[\pi_{t}^{nn}]$}.

Proof in Appendix

Using Lemma 2, we define the Nash equilibrium of the information sharing game in the following proposition:

**Proposition 3** *When emission taxes are the policy instrument in use, then with unknown common demand:*

#### In the Nash equilibrium each pair does not share information

Proposition 3 states exactly the opposite of Proposition 1. Now, the governments and the firms do not reach an agreement since the firms are unwilling to reveal their private information about θ. That is because in the case that the firms supply their information to the governments they will adjust the tax accordingly. For example, if θ is positive, then the government raises the emissions tax further, while if θ is negative then the government cut the tax by $\frac{\theta g[3k+g(-1+2k)]}{\tau_{2}}$. This policy, however, is restrictive for a firm because it cannot exploit the benefits sourcing from the convexity of the profit function. Despite the fact that the expected profits are lower if a pair reaches an agreement the governments are still willing to accept information as suggested in Lemma 2. The reason is that pollution is less flexible in the complete information case and implies higher expected welfare which, in turn, outweighs the negative effect on expected welfare because of lower expected profits. Yet, the important feature in this case is that the firms prefer to keep private their information and this is sufficient to make an agreement impossible. In contrast to the case of standards, if taxes are implemented, the assumption of incomplete information should be taken into account in the strategic environmental policy models.

In order the results of this section to be comparable with those of the previous section we will see if the Nash equilibrium coincides with the optimal solution from the social perspective. Proposition 4 illustrates this comparison:

**Proposition 4** *When emission taxes are the policy instrument in use, then with unknown common demand:*

*Expected welfare under information sharing is higher than expected welfare when the two pairs do not share information, i.e*., $E[w_{t}^{cc}]>E[w_{t}^{nn}]$.

Proof in Appendix

Put it differently, Proposition 4 suggests that the Nash equilibrium with taxes is sub-optimal from the social perspective. The residents in the two countries would be better off if the firms and the governments share information, even though this does not happen in the Nash equilibrium of the game.

## 5. Concluding Remarks

In this paper we examine the issue of information sharing in a strategic trade model where the exporting firms yield a pollutant as a by-product of production. Environmental policy instruments, emission standards and taxes, instead of the traditional trade instruments are implemented. Similarly to CM, we examine whether the firms and the governments will reach an agreement concerning information sharing. Contrary to CM, our results suggest that when emission taxes are used the firms are unwilling to reveal information. As a result an agreement, although socially desirable, is not achieved as a Nash equilibrium despite the fact that the firms compete in quantities. The main contribution of this study is that not only the mode of competition of the firms matters, *i.e.*, price or quantity competition, but the mode of competition of the governments is equally important, *i.e.*, price or quantity instrument. In particular, the use of a price instrument daunts the participants from a possible agreement, while regulation through quantity constraints encourages all parties towards an agreement.

The suggestions of this study can be further extended. For instance, the fact that the goods may be consumed in the two exporting countries and thus consumer surplus is a determinant of welfare may affect the decisions of the governments concerning the level of regulation but not the decision to reach an agreement as long as the driving forces of the mechanism remain in place. This is true also if we allow for a higher number of firms or for pollution to be trans-boundary. If this is the case, again standards (taxes) will (not) induce the participants towards an agreement as in good times environmental policy will be laxer (tighter), while in bad times will be tighter (laxer). Therefore, the results are expected to differ only quantitatively since the damage in the case of trans-boundary pollution is expected to be higher. Another modification of the model concerns the mode of uncertainty. Firms might hold private information about their costs of abatement instead of the common demand. Put it differently, the governments do not know the exact level of abatement costs and, thus, they set up an agreement to gain the extra information. We shall expect again that, in the case of standards, contrary to that of taxes, information revelation exploits further the convexity of the profit function, enforcing a bilateral agreement. Even if the functional forms used are generalized such that an interior solution exists we expect that the basic implications of this study will be replicated, since the governments are expected to increase the variability of production through standards when the firms reveal information, while the implementation of taxes reduces the variability in production under complete information driving them to keep private their information.

## Appendix

### Solution for the Asymmetric Case with Standards

The problem is again solved by backwards induction. The reaction function of output of the domestic firm is still obtained by equation (6) and for the foreign firm is analogous. In Stage 3 the domestic government maximize welfare under complete information as given in (8), while the foreign one under uncertainty using the analogous equation of (8) after setting θ = 0. Solving the governments best response functions as well as calculating each government’s expectation of the rival reaction function, the other’s expectation of its own reaction function together with (7) and the corresponding equation for the foreign firm we get the Bayes Nash equilibrium:

(A1) $$\left\{\begin{array}{c} x^{cn}=\frac{\left(B-c)(1+g)(3+g)(g+k\right)}{\zeta_{2}}+\frac{{\left(1+g\right)}^{2}(3+g)(g+k)\theta }{\zeta_{1}}\\ X^{cn}=\frac{\left(B-c)(1+g)(3+g)(g+k\right)}{\zeta_{2}}+\frac{\zeta_{3}\theta }{\zeta_{1}}\\ z^{cn}=\frac{(B-c)g{\left(2+g\right)}^{2}}{\zeta_{2}}+\frac{(1+g)g{\left(2+g\right)}^{2}\theta }{\zeta_{1}}\\ Z^{cn}=\frac{(B-c)g{\left(2+g\right)}^{2}}{\zeta_{2}} \end{array}\right\}$$

where *ζ*_3_ = *g*[3 + *g*(3 + *g*)] + (1 + *g*)^2^ (3 + *g*)*k* and the superscript (cn) describes the fact that the domestic government and firm share information, while the foreign pair does not.

Given the equilibrium values for outputs and standards (A1) in the two countries we determine the expected profits and welfare levels in the two countries as follows:

(A2) $$E[\pi^{cn}]=E[\Pi^{nc}]=[(2+g)(g+k)\zeta_{1}+g^{2}{\left(2+g\right)}^{4}k]\left(\frac{{\left(B-c\right)}^{2}(2+g)}{2\zeta_{2}^{2}}+\frac{(2+g){\left(1+g\right)}^{2}}{2\zeta_{1}^{2}}\text{var}(\theta )\right)$$

(A3) $$E[\Pi^{cn}]=E[\pi^{nc}]=\frac{{\left(B-c\right)}^{2}(2+g)[g\zeta_{1}+{\left(1+g\right)}^{2}{\left(3+g\right)}^{2}k(g+k)]}{2\zeta_{2}^{2}}+\frac{(2+g)\zeta_{3}^{2}}{2\zeta_{1}^{2}}\text{var}(\theta )$$

(A4) $$E[w^{cn}]=E[W^{nc}]=\frac{{\left(B-c\right)}^{2}(2+g)(g+k)\zeta_{1}}{2\zeta_{2}^{2}}+\frac{(2+g)(g+k){\left(1+g\right)}^{2}}{2\zeta_{1}}\text{var}(\theta )$$

(A5) $$\text{and}\;E[W^{cn}]=E[w^{nc}]=\frac{{\left(B-c\right)}^{2}(2+g)(g+k)\zeta_{1}}{2\zeta_{2}^{2}}+\frac{(2+g)\zeta_{3}^{2}}{2\zeta_{1}^{2}}\text{var}(\theta )$$

where the superscript (nc) describes the fact that the domestic government and firm do not share information, while the foreign pair does.

### Proof of Lemma 1

Using equations (10), (11), (13), (14) and (A2)–(A5) we obtain the differences *E*[*w^cc^*] − *E*[*w^nc^*], *E*[*π^cc^*] − *E*[*π^nc^*], *E*[*w^cn^*] − *E*[*w^nn^*] and *E*[*π^cn^*] − *E*[*π^nn^*] as follows:

$$\left\{\begin{array}{c} E[w^{cc}]-E[w^{nc}]=\frac{\zeta_{4}}{2\zeta_{1}^{2}\zeta_{2}^{2}}\text{var}(\theta )>0,\\ E[\pi^{cc}]-E[\pi^{nc}]=E[w^{cc}]-E[w^{nc}]+\frac{g^{2}{\left(2+g\right)}^{4}k}{2\zeta_{2}^{2}}\text{var}(\theta )>0,\\ E[w^{cn}]-E[w^{nn}]=\frac{\left\{g[18+g(36+g(24+5g))]+2{\left(1+g\right)}^{2}{\left(3+g\right)}^{2}k\}g^{2}{\left(2+g\right)}^{3}(g+k\right)}{2\zeta_{1}^{2}\zeta_{2}^{2}}\text{var}(\theta )>0,\\ E[\pi^{cn}]-E[\pi^{nn}]=\frac{g^{2}{\left(2+g\right)}^{4}k[\zeta_{1}+{\left(1+g\right)}^{2}{\left(3+g\right)}^{2}k]}{2{\left(3+g\right)}^{2}\zeta_{1}^{2}}\text{var}(\theta )>0. \end{array}\right\}$$

where:

*ζ*_4_ = *g*^2^(2 + *g*)^4^{*g*^3^(1 + *g*)[81 + *g*(171 + *g*(143 + *g*(55 + 8*g*)))] + *g*^2^[9 + 2*g*(8 + *g*(5 + *g*))] [27 + 2*g*(28 + *g*(17 + 3*g*))]*k* + *g*(1 + *g*)^2^(3 + *g*)^2^[27 + 2*g*(26 + *g*(16 + 3*g*))]*k*^2^ + (1 + *g*)^4^(3 + *g*)^4^*k*^3^}.

Q.E.D.

### Proof of Proposition 2

Using equations (11) and (14) we obtain the difference *E*[*w^cc^*] − *E*[*w^nn^*] as follows:

$$E[w^{cc}]-E[w^{nn}]=\frac{g^{3}{\left(2+g\right)}^{3}[g(5+2g)+{\left(3+g\right)}^{2}k]}{2{\left(3+g\right)}^{2}\zeta_{2}^{2}}\text{var}(\theta )>0$$

Q.E.D.

### Solution for the Asymmetric Case with Taxes

The reaction function of output of the domestic firm is given by equation (15) and for the foreign firm is analogous. In Stage 3 the domestic government maximize welfare under complete information as given in (18), while the foreign one under uncertainty using the analogous equation of (18) after setting θ = 0. Solving the governments best response functions as well as calculating each government’s expectation of the rival reaction function, the other’s expectation of its own reaction function together with (17) and the corresponding equation for the foreign firm we get that the Bayes Nash equilibrium:

(A6) $$\left\{\begin{array}{c} x_{t}^{cn}=\frac{\left(B-c)(3+2g)(g+k\right)}{\tau_{2}}+\frac{(3+2g)(g+k)\theta }{\tau_{1}}\\ X_{T}^{cn}=\frac{\left(B-c)(3+2g)(g+k\right)}{\tau_{2}}+\frac{\tau_{3}\theta }{\tau_{2}}\\ t^{cn}=\frac{\left(B-c)g[3k+g(-1+2k)\right]}{\tau_{2}}+\frac{g[3k+g(-1+2k)]\theta }{\tau_{1}}\\ T^{cn}=\frac{\left(B-c)g[3k+g(-1+2k)\right]}{\tau_{2}} \end{array}\right\}$$

where *τ*_3_ = *g*(3 + *g*) + (1 + *g*)(3 + 2*g*)*k*.

Given the equilibrium values for outputs and taxes (A6) in the two countries we can determine the expected profits and welfare levels in the two countries as follows:

(A7) $$E[\pi_{t}^{cn}]=E[\Pi_{T}^{nc}]=(2+g)[g^{2}(9+8g)+6g(3+2g)k+{\left(3+2g\right)}^{2}k^{2}]\left(\frac{{\left(B-c\right)}^{2}}{2\tau_{2}^{2}}+\frac{\text{var}(\theta )}{2\tau_{1}^{2}}\right)$$

(A8) $$E[\Pi_{T}^{cn}]=E[\Pi_{t}^{nc}]=\frac{g[3k+g(-1+2k)]\{27k+g[39+22g+4(6+g)k]\}}{18\tau_{2}\tau_{3}}\text{var}(\theta )$$

(A9) $$E[w_{t}^{cn}]=E[W_{T}^{nc}]=\frac{{\left(B-c\right)}^{2}(2+g)[g^{2}(9+8g)+6g(3+2g)k+{\left(3+2g\right)}^{2}k^{2}]}{2\tau_{2}^{2}}+\frac{\tau_{3}^{2}}{\tau_{1}^{2}}\text{var}(\theta )$$

(A10) $$\text{and}\;E[W_{T}^{cn}]=E[w_{t}^{nc}]=\frac{{\left(B-c\right)}^{2}(2+g)(g+k)\tau_{1}}{2\tau_{2}^{2}}-\frac{(k-2)\tau_{3}^{2}}{2\tau_{1}^{2}}\text{var}(\theta )$$

### Proof of Lemma 2

Using equations (20), (21), (23), (24) and (A7)–(A10) we obtain the differences $E[w_{t}^{cc}]-E[w_{t}^{nc}]$, $E[\pi_{t}^{cc}]-E[\pi_{t}^{nc}]$, $E[w_{t}^{cn}]-E[w_{t}^{nn}]$ and $E[\pi_{t}^{cn}]-E[\pi_{t}^{nn}]$ as follows:

$$\left\{\begin{array}{c} E[w_{t}^{cc}]-E[w_{t}^{nc}]=\frac{{\left(g-3k-2gk\right)}^{2}\tau_{4}}{18\tau_{1}^{2}\tau_{2}^{2}}\text{var}(\theta )>0,\\ E[\pi_{t}^{cc}]-E[\pi_{t}^{nc}]=E[\pi^{cn}]-E[\pi^{nn}]-\frac{g{\left[(3k+g(-1+2k)\right]}^{2}\tau_{5}}{18\tau_{1}^{2}\tau_{2}^{2}}\text{var}(\theta )<0,\\ E[w_{t}^{cn}]-E[w_{t}^{nn}]=\frac{{\left(g-3k-2gk\right)}^{2}}{18\tau_{1}}\text{var}(\theta )>0,\\ E[\pi^{cn}]-E[\pi^{nn}]=-\frac{g[3k+g(-1+2k)]\{45k+g[81+54k+8g(5+2k)]\}}{18\tau_{1}^{2}}\text{var}(\theta )<0. \end{array}\right\}$$

where:

*τ*_4_ = *g*^3^{81 + 2*g*[72 + *g*(40 + 7*g*)]} + *g*^2^{243 + *g*[612 + *g*(554 + 11*g*(20 + 3*g*))]}*k* + *g*(3 + 2*g*){81 + 2*g*[105 + *g*(96 + *g*(37 + 5*g*))]}*k*^2^ + (1 + *g*)^2^ (3 + *g*)^2^ (3 + 2*g*)^2^ *k*^3^
τ_5_ = *g*^2^[1134 + *g*(1161 + 298*g*)] + 2*g*(3 + 2*g*)[270 + *g*(261 + 62*g*)]*k* + (3 + 2*g*)^2^ [54 + 5*g*(9 + 2*g*)]*k*^2^

Q.E.D.

### Proof of Proposition 4

Using equations (21) and (24) we obtain the difference $E[w_{t}^{cc}]-E[w_{t}^{nn}]$ as follows:

$$E[w_{t}^{cc}]-E[w_{t}^{nn}]=\frac{\left[3k+g(-1+2k)]\{g^{2}(27+14g)+g[54+7g(6+g)]k+{\left(3+g\right)}^{2}(3+2g)k^{2}\right\}}{18\tau_{2}^{2}}\text{var}(\theta )>0$$

Q.E.D.
